# Cohort study of standardized treatment pathways by multidisciplinary tumor board decision making in patients with salivary gland malignancies

**DOI:** 10.1002/ijc.70382

**Published:** 2026-02-14

**Authors:** Nils Feldmann, Gunnar Wichmann, Andreas Dietz, Markus Pirlich

**Affiliations:** ^1^ Department of Otolaryngology, Head and Neck Surgery University Leipzig Leipzig Germany

**Keywords:** multidisciplinary tumor board, cohort study, neoplasm metastasis, practice guidelines as topic, quality assurance, health care, salivary gland neoplasms

## Abstract

Salivary gland malignancies (SGM) are heterogeneous regarding origin, biology and prognosis. We investigated patterns of clinical and pathological features and outcome differences attributable to standardized decision making for treatment pathways before and after establishing our multidisciplinary tumor board (MDTB) in 2007. We retrospectively analyzed data of electronic health records, the Saxon cancer registry and the clinic's tumor database (1990–2024). Statistical analysis included contingency tables, *Pearson's* chi‐squared tests for categorical, *Student's t* test for numerical data, Kaplan–Meier cumulative survival plots and log‐rank tests for time‐dependent outcome measures. Among 200 patients, 83 were diagnosed before and 117 since 2007. Treatment modalities included surgery in 93% of cases, with 33.5% receiving adjuvant radiotherapy and 12.5% receiving adjuvant chemo‐radiotherapy. We observed disease progression in 50% of patients. Locoregional free survival was superior since 2007 (*p* = .035). Distant metastasis‐free survival (DMFS) decreased related to earlier detection of distant metastasis (M1) linked to prevention of deaths from the index cancer in patients with M1 at diagnosis or relapse (rM1) since 2007 (*p* = .120). Moreover, conditional OS was not affected for patients with M1/rM1 diagnosis by numerically increased detection of distant metastasis since 2007 (*p* = .574). MDTB decision making at a certified cancer center is accompanied by increasing numbers of diagnosis of SGM and better locoregional control but not DMFS due to higher metastasis detection rate and treatment. Despite excellent and numerically improved disease‐specific survival, 10‐years OS remained similar.

## Graphical abstract

What's new?

The overall survival of patients with salivary gland malignancies has shown limited progress. Here, the authors evaluated the impact of establishing a multidisciplinary tumor board to ensure standardized diagnostic and treatment procedures at a certified cancer center. The retrospective analysis of two cohorts of patients diagnosed before and after the establishment of the tumor board shows improved locoregional relapse‐free survival and successful prevention of cancer‐related deaths with multidisciplinary decision making. While the implementation of [18F]‐FDG‐positron‐emission tomography/computer tomography also led to accurate distant metastasis diagnostics, the distant metastasis‐free survival nominally worsened despite better disease‐specific care, with the 10‐year overall survival remaining stagnant.

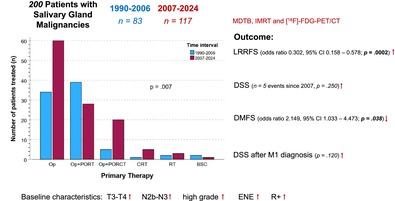

**Abbreviations**
ACC: adenoid cystic carcinoma
ChT: chemotherapy
CRD: cancer‐related death
CT: computer tomography
DM: aka rM1 distant metastasis
DMFS: distant metastasis‐free survival
DSS: disease‐specific survival
FDG: fluorodesoxyglucose
HNSCC: head and neck squamous cell carcinomas
IMRT: intensity‐modulated radiotherapy
LR: aka rT local relapse
LRFS: local relapse‐free survival
LRR: locoregional relapse
LRRFS: locoregional relapse‐free survival
MDTB: multidisciplinary tumor board
MPT: malignant parotid tumors
NCRD: non‐cancer‐related death
NCRS: non‐cancer‐related survival
ND: neck dissection
NR: aka rN nodal relapse
NRFS: nodal relapse‐free survival
Op: surgery
OS: overall survival
PET: positron‐emission tomography
PFS: progression‐free survival
PORT: postoperative radiotherapy
RT: radiotherapy
SGM: salivary gland malignancies
TI: time interval

## 1 INTRODUCTION

Salivary gland malignancies (SGM) are a very rare and heterogeneous group of carcinomas.^1^ Epidemiological data differ depending on their geographical region and the clinics that diagnosed and treated them^2^ In total, together with further malignant entities, SGM define a group of at least 21 histological subtypes and account for approximately 5% of all head and neck tumors.

In case of clinically suspected SGM, diagnostic imaging is performed using ultrasound, computer tomography (CT) or magnetic resonance imaging (especially for tumors suspicious for perineural, skin, bone or vascular infiltration) and positron‐emission tomography (PET)/CT (especially for suspected distant metastasis).^3^, ^4^, ^5^, ^6^ Histopathologic confirmation is mandatory and additional immunohistochemical examinations enable a more reliable diagnosis and classification with consequences for choosing appropriate treatment.^4^, ^5^, ^6^, ^7^

Nowadays, decision making for SGM takes place between standardization and personalization and should be orientated at guidelines and current scientific evidence. It is well established that a multidisciplinary tumor board (MDTB) should discuss the optimal treatment strategy.^8^, ^9^

With the introduction of a “head and neck”‐MDTB at University Hospital Leipzig from 2007 onwards, we can ensure standardized diagnostics and treatment uniformity regarding treatment modalities. Due to the limited progress in overall survival (OS) of SGM in recent years, the aim of this study was to evaluate the guideline‐oriented decision making in MDTB about diagnostic procedures and the thereafter applied treatment regimen and their joint impact on overall (OS), disease‐specific (DSS), non‐cancer‐related (NCRS), progression‐free (PFS), local relapse‐free (LRFS), nodal relapse‐free (NRFS), locoregional relapse‐free (LRRFS), and distant metastasis‐free survival (DMFS). We were interested in changing distributions of case numbers over time and potential effects from quality assurance attempts. These attempts included standardization of diagnostic processes and therapy of SGM subtypes according to standardized operating procedures along the introduction of [^18^F]‐fluorodesoxyglucose (FDG) PET/CT, intensity‐modulated radiotherapy (IMRT), and establishing the MDTB. Consequently, we analyzed changing patterns of applied diagnostics and therapies and the related outcome.

## 2 MATERIALS AND METHODS

This study employed a retrospective, observational, non‐interventional, cross‐sectional design. We identified 359 patients with malignant salivary gland tumors diagnosed between 1990 and 2024 at our clinic using the internal documentation system of the University Hospital Leipzig (SAP), the Saxon Cancer Registry, and the clinic's (Clinic and Polyclinic for ENT Medicine/Plastic Surgery) internal tumor database. From 2013 onwards, proprietary software (OncoFunction) was additionally available, which included data on functional outcomes and contributed to the documentation of follow‐up data. Raw data from the ENT clinic's internal database was completed and researched in the documentation system of Leipzig University Hospital (SAP). In addition to the patient's basic data, all cancer‐specific and death‐related information from doctors' letters, pathology reports, tumor board conferences, outpatient cards, inpatient stays, etc. were documented. Those core variables included for final data analysis are shown in Table 1.

**TABLE 1:** Baseline characteristics of the study population. Distributions are shown with number of cases and percentage in brackets.

		Total		1990–2006		2007–2024		
		*n*	(%)	*n*	(%)	*n*	(%)	*p*‐Value
		200	(100.0)	83	(41.5)	117	(58.5)	
Sex	Male	119	(59.5)	45	(54.2)	74	(63.2)	*p* = .200
	Female	81	(40.5)	38	(45.8)	43	(36.8)	
Age	<50 years	43	(21.5)	16	(19.3)	27	(23.1)	*p* = .322
	≥50–59 years	44	(22.0)	20	(24.1)	24	(20.5)	
	≥60–69 years	37	(18.5)	13	(15.7)	24	(20.5)	
	≥70–79 years	55	(27.5)	28	(33.7)	27	(23.1)	
	≥80 years	21	(10.5)	6	(7.2)	15	(12.8)	
Localization	C07	124	(62.0)	64	(77.1)	60	(51.3)	***p* < .001**
	Other	76	(38.0)	19	(22.9)	57	(48.7)	
Histology	8070_PEC	58	(29.0)	26	(31.3)	32	(27.4)	***p* = .031**
	8140_AdC	18	(9.0)	7	(8.4)	11	(9.4)	
	8147_Bad	5	(2.5)	0	(0.0)	5	(4.3)	
	8200_ACC	42	(21.0)	17	(20.5)	25	(21.4)	
	8430_Muk	22	(11.0)	7	(8.4)	15	(12.8)	
	8500_SGC	9	(4.5)	2	(2.4)	7	(6.0)	
	8550_AciCC	20	(10.0)	9	(10.8)	11	(9.4)	
	8562_EpM	3	(1.5)	1	(1.2)	2	(1.7)	
	8941_exP	11	(5.5)	9	(10.8)	2	(1.7)	
	8982_Myo	7	(3.5)	5	(6.0)	2	(1.7)	
	MASC	5	(2.5)	0	(0.0)	5	(4.3)	
Grading	High grade	45	(22.5)	8	(9.6)	37	(31.6)	***p* < .001**
	Other	155	(77.5)	75	(90.4)	80	(68.4)	
TNM version	5	50	(25.0)	50	(60.2)	0	(0.0)	***p* < .001**
	6	47	(23.5)	32	(38.6)	15	(12.8)	
	7	60	(30.0)	1	(1.2)	59	(50.4)	
	8	43	(21.5)	0	(0.0)	43	(36.8)	
T category	T0	4	(2.0)	2	(2.4)	2	(1.7)	*p* = .071
	T1	41	(20.5)	16	(19.3)	25	(21.4)	
	T2	72	(36.0)	39	(47.0)	33	(28.2)	
	T3	44	(22.0)	11	(13.3)	33	(28.2)	
	T4a	33	(16.5)	13	(15.7)	20	(17.1)	
	T4b	6	(3.0)	2	(2.4)	4	(3.4)	
N category	N0	127	(63.5)	60	(72.3)	67	(57.3)	*p* = .137
	N1	29	(14.5)	11	(13.3)	18	(15.4)	
	N2a	2	(1.0)	1	(1.2)	1	(0.9)	
	N2b	29	(14.5)	9	(10.8)	20	(17.1)	
	N2c	6	(3.0)	2	(2.4)	4	(3.4)	
	N3	7	(3.5)	0	(0.0)	7	(6.0)	
M category	M0	187	(93.5)	79	(95.2)	108	(92.3)	*p* = .417
	M1	13	(6.5)	4	(4.8)	9	(7.7)	
ENE	No ENE	176	(88.0)	70	(84.3)	106	(90.6)	***p* < .001**
	ENE	8	(4.0)	0	(0.0)	8	(6.8)	
	Unknown	16	(8.0)	13	(15.7)	3	(2.6)	
UICC stage	I	33	(16.5)	13	(15.7)	20	(17.1)	*p* = .065
	II	55	(27.5)	32	(38.6)	23	(19.7)	
	III	40	(20.0)	13	(15.7)	27	(23.1)	
	IVA	49	(24.5)	19	(22.9)	30	(25.6)	
	IVB	10	(5.0)	2	(2.4)	8	(6.8)	
	IVC	13	(6.5)	4	(4.8)	9	(7.7)	
Therapy	BSC	3	(1.5)	2	(2.4)	1	(0.9)	***p* = .007**
	RT	5	(2.5)	2	(2.4)	3	(2.6)	
	CRT	6	(3.0)	1	(1.2)	5	(4.3)	
	Op	94	(47.0)	34	(41.0)	60	(51.3)	
	Op + PORT	67	(33.5)	39	(47.0)	28	(23.9)	
	Op + PORCT	25	(12.5)	5	(6.0)	20	(17.1)	
Surgery	Op (Op, Op + PORT, Op + PORCT)	186	(93.0)	78	(94.0)	108	(92.3)	*p* = .649
	No surgery	14	(7.0)	5	(6.0)	9	(7.7)	
Type of surgery	Complete parotidectomy	54	(27.0)	24	(28.9)	30	(25.6)	***p* = .028**
	Partial parotidectomy	70	(35.0)	37	(44.6)	33	(28.2)	
	Complete submandibulectomy	16	(8.0)	7	(8.4)	9	(7.7)	
	Partial submandibulectomy	4	(2.0)	1	(1.2)	3	(2.6)	
	Other surgery	41	(20.5)	8	(9.6)	33	(28.2)	
	None	15	(7.5)	6	(7.2)	9	(7.7)	
Residual tumor	R0	153	(82.3)	69	(89.6)	84	(77.1)	***p* = .047**
	R1	29	(15.6)	6	(7.8)	23	(21.1)	
	R2	4	(2.2)	2	(2.6)	2	(1.8)	
Neck dissection	Bilateral selective ND	6	(3.0)	2	(2.4)	4	(3.4)	*p* = .158
	Bilateral SND + RND	5	(2.5)	0	(0.0)	5	(4.3)	
	Unilateral radical ND	8	(4.0)	4	(4.8)	4	(3.4)	
	Unilateral selective ND	40	(20.0)	15	(18.1)	25	(21.4)	
	Bilateral elective ND	9	(4.5)	1	(1.2)	8	(6.8)	
	Unilateral elective ND	48	(24.0)	24	(28.9)	24	(20.5)	
	No ND	84	(42.0)	37	(44.6)	47	(40.2)	

Abbreviations: BSC, best supportive care; CRT, concurrent radiochemotherapy; ENE, extranodal extension; MASC, mammary analogue secretory carcinoma; ND, neck dissection; Op, surgery only; Op + PORCT, surgery followed by radiochemotherapy; Op + PORT, surgery followed by postoperative radiotherapy; SND, selective neck dissection; RCht, radiochemotherapy; RND, radical neck dissection; RT, radiotherapy; TNM, tumor, nodus, metastasis; UICC, union internationale contre le cancer.

*Note*: The bold values indicate *p*<0.05.

**FIGURE 1:**
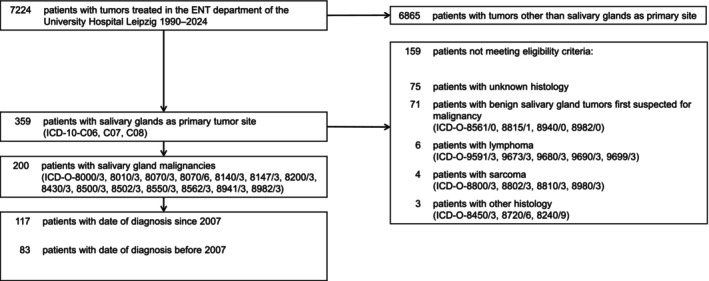
CONSORT diagram showing the selection process of patients with salivary gland tumors as well as the number of patients diagnosed before and since 2007.

Inclusion criteria were malignant tumors classified according to the origin of the primary encoded as international classification of diseases (ICD)‐10‐C06, C07, and C08. We have linked our dataset with the national health system ID as the encounter variable to the data of the Saxon Cancer Registry. Twenty‐six patients and nine tumors were not found as they were registered elsewhere and excluded. Only congruent patient records were included in the studies. For another 40 patients, neither we nor the cancer registry could find sufficient information specifying the histological subtype of the tumor. Head and neck squamous cell carcinomas (HNSCC) were included, as long as the pathologist described the tumor as a primary SGM. Benign neoplasms first suspected to be malignant, malignancies other than salivary glands as primary site, lymphomas, sarcomas, and inadequately documented tumors were excluded. After applying inclusion and exclusion criteria, the final study population comprised 200 patients (Figure 1; Table 1). There were no restrictions regarding disease severity, age spectrum, nor previous therapies. Patients with severe comorbidities or cooperation problems were not excluded and pregnant women not present. The study followed a parallel group design to test for differences between cohorts based on time of first diagnosis of SGM before 2007 (time interval 1 [TI1]) and since 2007 (time interval 2 [TI2]).

### 2.1 Standardized diagnostic workup and treatment since 2007

As recommended, the treatment of SGM was primarily surgical (Op)^4^, ^5^, ^6^, ^10^, ^11^ as only if the tumor is not resectable it should be irradiated (RT).^7^, ^8^, ^9^, ^12^ For malignant parotid tumors (MPT) classified as T1–T2 and as low‐grade, featuring no risk factors, partial parotidectomy should be the surgery of choice.^10^ In case of T3–T4 or high‐grade MPT, surgeons contemplate total or subtotal parotidectomy.^10^ Tumors of the submandibular gland, the sublingual gland and the minor salivary glands were usually resected completely en bloc if nerve preservation in particular allowed complete glandectomy. Lymph nodes were removed in the case of a cN+ situation and a primary surgical treatment decision was made in the MDTB.^13^ Only in a few patients was the neck dissection (ND) extended up to radical ND.^13^ If the tumor had risk factors for lymph node metastasis, lymph nodes at risk were removed preventively (elective ND).^14^

Risk factor‐adapted postoperative radiotherapy (PORT) was applied to adenoid cystic carcinoma (ACC), T3‐T4, high‐grade tumors as well as to all tumors with postoperative residual tumor detectable micro‐ (R1) or macroscopically (R2), with perineural (Pn1), lymphatic (L1), or intravenous (V1) infiltration and whenever disease‐positive neck nodes (N+) are present as these risk factors increase the risk for local and/or nodal recurrence.^12^ In the presence of T3/T4 and/or high‐grade tumors, the neck was RT despite being cN0 or confirmed pN0 category (elective neck irradiation).^15^ According to guidelines, patients undergoing PORT should not receive additional chemotherapeutic treatment (ChT) outside of clinical trials.^16^, ^17^ Moreover, ChT is not indicated for primary non‐surgical or adjuvant treatment concepts.^16^, ^17^ If the MDTB decided on ChT, cisplatin (up to three cycles of 100 mg/m^2^ day 1; 22; 43) was recommended.^16^, ^17^

Systemic therapy was considered for metastatic or recurrent SGM whenever Op and/or RT were deemed to be either not possible according to functionality or probably not successful regarding achievement of clear margins R0 ≥ 5 mm.^16^, ^17^ Molecular pathological analysis potentially revealing targetable molecular alterations indicates targeted therapy using small molecules.^18^ If, after comprehensive molecular analysis, no molecular targets were detected that could have been addressed with approved inhibitors, polyChT was used.^16^, ^17^ However, as the response to systemic treatment is mostly limited and therefore only recommended for SGM without curative treatment options, especially targeted therapy was rarely used (*n* = 4). This prevented subgroup analyses.

In 2006, a [^18^F]‐FDG‐PET/CT became available and allowed for extended diagnostic workup, while IMRT was available for every head and neck cancer patient in need of radiotherapy including those with SGM. Enhanced planning capabilities for standardized irradiation dosage according to published evidence were utilized to apply volume‐defined irradiation while sparing dosage for particular structures at risk, including parotid sparing dose distribution to preserve at least one parotid with dosage below 25 Gy to prevent xerostomia and ensure acceptable quality of life.

Moreover, we established in 2007 our MDTB that since then met weekly on a regular basis for consented decision making for the individually probably best diagnostic approach and treatment option for the patient. By careful and well‐standardized pathologic examination benefiting from advances in the field, the MDTB defined standardized diagnostic pathways to follow. The MDTB considered the extension of the primary tumor (and categorization according to T categories), neck nodes (categorized in N categories), and distant metastasis (M category) summarized into UICC status but even more the knowledge about molecular features, response to various treatment modalities, etc. Consequently, treatment equality was ensured from 2007 onwards via implementation of the MDTB with pre‐, intra‐, and post‐treatment presentation of all patients to ensure adherence to standardized pathways. Follow‐up was standardized (3‐month interval for 3 years, thereafter every 6 months).

### 2.2 Statistical analysis

We calculated absolute and relative frequencies and determined measures of central tendency and dispersion. We employed calculation of mean and 95% confidence intervals (CI), contingency tables (cross‐tables), *Pearson's* chi‐square (*χ* ^2^) test for categorical variables, and *Student's t* tests for numerical data. Prespecified time‐to‐event data analysis was performed by the Kaplan–Meier method for estimating survival probabilities regarding OS, DSS, NCRS, LRFS, LRRFS, NRFS, and DMFS. The starting point was the date of the panendoscopy and biopsy leading to the diagnosis of the histologic subtype, and the recorded TI was calculated up to confirmation of the event of interest. The events of interest were death from any cause for OS, death from the index cancer only for DSS, or death from other cause than the index cancer for NCRS. Diagnosis of progressing disease (according to response evaluation criteria in solid tumors (RECIST) 1.1 criteria) or any type of relapse (local [LR aka rT], nodal [NR aka rN], locoregional [LRR], or distant failure [DM aka rM1 distant metastasis]) or death from the index cancer defined progression and thus an event of interest regarding PFS, which led to patients being removed from the “at risk” group in PFS analysis, regardless of whether they subsequently experienced another event of this type or not. LRFS is the time between diagnosis and the date of pathohistologic confirmation of local recurrence, rpT. NRFS is the time between diagnosis to the date of pathohistologic confirmation of nodal recurrence, rpN. LRRFS is the time of diagnosis unto the date of rpT or rpN, whichever of the events (rpT or rpN) occurred first (see PFS). DMFS was calculated from the difference between the date of confirmed rM1 and the date of diagnosis. For each of the different survival time analyses, patients alive and without event of interest were right‐censored at the date of the last visit. Log‐rank tests were used for comparing survival data. For comparing means, we used parametric *t* tests. Survival time was defined as the time from date of diagnosis to the occurrence of a defined event (cancer‐related death (CRD), non‐cancer‐related death (NCRD), LR, LRR, NR, or DM) by right censoring of patients without the particular event at last examination or end of clinical follow‐up for patients alive at 120 months.

Following these analyses, we performed further post hoc analyses on conditional OS, DSS, and NCRS after M1/rM1 diagnosis applying Kaplan–Meier plots and log‐rank tests to aid interpretation. Those patients who were diagnosed with M1/rM1 before 2007 were compared with those who were diagnosed with M1/rM1 since 2007. In these analyses the time of initial diagnosis of the primary tumor was not of prime interest as rather the question about availability whether or not of advanced diagnostics including [^18^F]‐FDG‐PET/CT at the time of diagnosis of distant metastasis was the focus here. Otherwise, all subgroup analyses were performed using the same methods as outlined above.

All statistical analyses were carried out using the IBM statistical software package for social sciences (IBM SPSS Statistics version 29).

## 3 RESULTS

### 3.1 Baseline characteristics

Analyses from contingency tables with *p‐values* calculated with *Pearson's* chi square (*χ* ^2^) test can be seen in Table 1.

**FIGURE 2:**
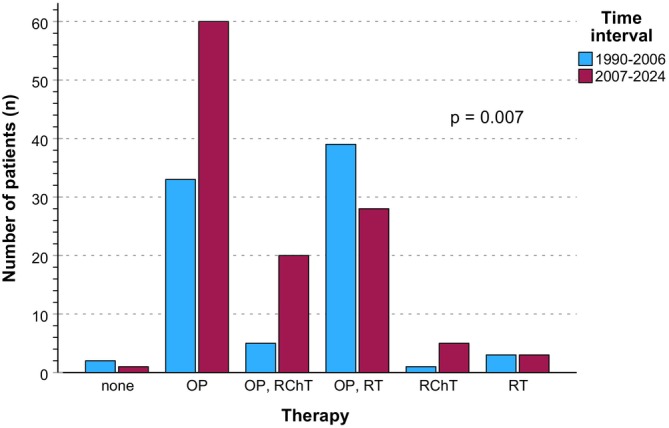
Bar charts illustrating absolute frequencies of primary treatment modalities in the strata 1990–2006 versus 2007–2024 and the *p*‐value from *Pearson's* chi‐square test. Modalities of primary therapy overall: (Op) surgery only; (RT) radiotherapy only; (Op + PORT) surgery followed by postoperative radiotherapy; (Op + PORCT) surgery followed by radiochemotherapy; and (CRT) concurrent radiochemotherapy.

Age migration can be seen in the group of patients aged 70–79 years, especially. They accounted for a larger proportion before 2007. This is accompanied by an increase in the number of patients aged 60–69 years, aged younger than 50 years, and those older than 79. The group of 50‐ to 59‐year‐old patients, on the other hand, has decreased after 2007 (Table 1). Since 2007, we have observed an increase in SGM of the minor salivary glands (RR parotids in TI2 0.6452, 95% CI [0.516–0.8066]; *p* < .001) and, associated with this, more excisions that are not partial or total glandulectomies have been performed. Furthermore, more tumors with maximum diameter between 4 and 6 cm or expanding extraparenchymally (T3) have been diagnosed. In addition, tumors feature more multiple ipsilateral (N2b) and bilateral (N2c) lymph node metastases as well as lymph node metastases larger than 6 cm (N3) since 2007. This led to more decisions for radical and bilateral ND and PORCT. This was accompanied by elevated diagnosis of poor differentiation (G3 and G4) and ENE and microscopic invasion (R1) in resection margins. As ENE became more frequent and complete excision (R0) was less frequently possible or refused considering functional outcome, especially PORCT (RR PORCT in TI2 3.487, 95% CI [1.361–8.937]; *p* = .006) was considered and implemented as a viable treatment option more often. ENE and R1 heighten the risk for locoregional relapse (LRR), and assuming that they can be addressed via extended adjuvant treatment concepts shifted therapy patterns overall (Table 1, Figure 2).

### 3.2 Survival analysis

The number of tumors diagnosed before and since 2007 has increased from 83 to 117 (Table 1). Since 2007, locoregional relapse‐free survival (LRRFS) was significantly improved (RR LRR in TI2 0.302, 95% CI [0.158–0.578]; *p* = .0002). Beside the increased number of tumors with risk factors, standardized diagnostic and therapeutic algorithms and higher sensitivity of imaging led to significantly higher detection of distant metastasis (RR DM in TI2 2.149, 95% CI [1.033–4.473]; *p* = .038) and consequently adjuvant systemic therapy treatment options were applied more often (Figure 2).

**FIGURE 3:**
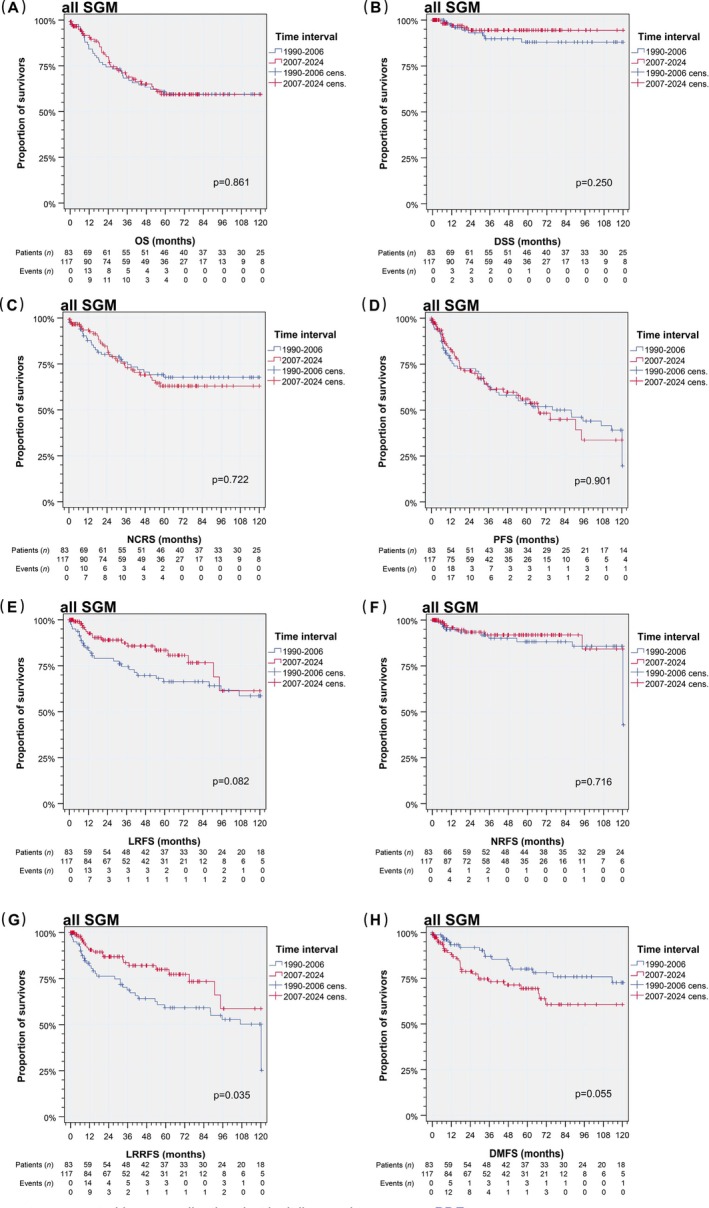
Kaplan–Meier plots and *p* values from log‐rank tests demonstrate deviating outcomes among 200 patients with salivary gland tumors according to date of diagnosis before and since 2007. (A) Overall survival (OS); (B) disease‐specific survival (DSS); (C) non‐cancer‐specific survival (NCRS); (D) progression‐free survival (PFS); (E) local relapse‐free survival (LRFS); (F) nodal relapse‐free survival (NRFS); (G) locoregional relapse‐free survival (LRRFS); (H) distant metastasis‐free survival (DMFS). SGM, salivary gland malignancies. [Correction added on 11 April 2026, after first online publication: Figure 3 has been replaced.].

According to Kaplan–Meier analysis (Figure 3), neither OS nor NCRS improved since 2007. However, the median survival times for all SGM together were above 120 months. Between 1990 and 2006, we detected eight CRD. Despite more frequent detection of M1/rM1 since 2007, the DSS was remarkably high as only five CRD (and only three CRD accompanied by M1/rM1) were documented. The intensified tumor‐specific care and closer follow‐up, including prescription of [^18^F]‐FDG‐PET/CT imaging in case of suspect locoregional findings, was identified as main contributor to elevated detection rates of M1 and PFS events also at an earlier stage of disease. In line with this, median PFS since 2007 was found to be slightly inferior to median PFS before 2007 (88.1, 95% CI 47.9–128.3 months in TI1 vs. 67.6, 95% CI 46.1–89.0 months in TI2; *p* = .901).

LRFS and NRFS clearly discriminate in TI comparison, but did not manage to withstand log‐rank test (Figure 3). Taking together local and nodal events, locoregional control since 2007 is superior (neuroendocrin neoplasm (NOE) LRR in TI1 = 37 (44.6%) vs. NOE LRR in TI2 = 25 (21.4%); *p* = .035, Figure 3). In sharp contrast to LRRFS especially, we observed worse DMFS since 2007 (NOE DM in TI1 = 16 (19.3%) vs. NOE DM in TI2 = 29 (24.8%); *p* < .001; Figure 3).

**FIGURE 4:**
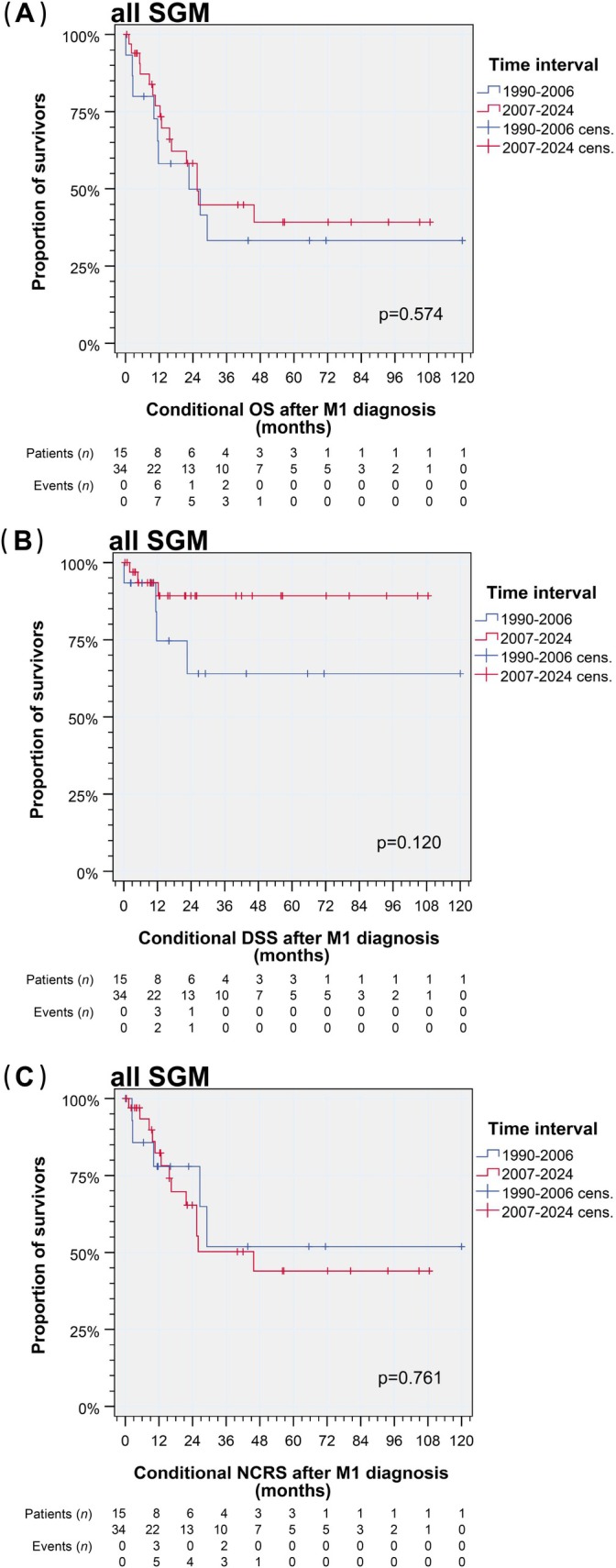
Kaplan–Meier plots and *p* values from log‐rank tests demonstrate deviating outcomes among 49 patients with salivary gland tumors according to date of M1/rM1 diagnosis before and since 2007. (A) Conditional overall survival (OS); (B) conditional disease‐specific survival (DSS); (C) conditional non‐cancer‐related survival (NCRS). SGM, salivary gland malignancies. [Correction added on 10 April 2026, after first online publication: Figure 4 has been replaced.].

However, earlier detection of distant metastasis since 2007 prevented deaths related to the index cancer, especially in patients with M1/rM1 diagnosis (Figure 4). The numerically increased detection of distant metastasis since 2007 did not affect NCRS of this particularly vulnerable group of patients with M1/rM1 diagnosis, and even conditional OS after detection of M1/rM1 was not affected (Figure 4).

## 4 DISCUSSION

The retrospective analyses of the two cohorts of SGM patients treated at the University Hospital Leipzig until 2006 and since 2007 revealed valuable insights into the changes of diagnostic procedures and applied therapies but also the changing landscape of SGM patients.

### 4.1 Increasing case numbers

Epidemiological data can be interpreted within world‐ and nationwide variations and our data aligns with the epidemiological characteristics described in the literature.^2^, ^19^, ^20^ We have seen increasing SGM case numbers since 2007 at the University Hospital Leipzig. The certification of the ENT clinic as a cancer center for head and neck carcinomas, including a dedicated salivary gland consultation service and proven expertise in salivary gland tumors, has resulted in a constant rise in referrals from throughout Germany. The center's incidence of SGM in year‐to‐year comparison therefore, based on the selective referral theory, has to be evaluated with caution. Accordingly, the rise in case numbers can be interpreted within the context of the rising number of referrals due to the center's reputation and not as a general incidence trend, although this cannot be completely ruled out based on our data. Notably, most of the rise is due to the rising number of small SGMs, which argues against selective referral as the only reason behind increased case numbers.

### 4.2 Changing patterns in diagnostics and treatment of SGM by MDTB decision making at a certified cancer center

Multidisciplinary, evidence‐based and individual decision making in tumor boards at certified cancer centers with the best diagnostic and therapeutic options and evidence‐based follow‐up intervals optimize patient outcomes for many tumor entities. This applies to HNSCC explicitly.^9^ Since 2007, we have mainly been able to ensure successful therapeutic intervention for the whole spectrum of SGM predominantly by applying surgery followed by risk factor‐adapted radio‐ or radiochemotherapy (Table 1, Figure 2). Enhanced pathohistologic workup, standardized procedures and automatization of diagnostic processes plus antibody‐ and biomarker‐based sub‐classification of SGM have substantially contributed to forming a reliable basis for evidence‐based decision making for further required diagnostics by, for example, radiologic imaging and guiding risk‐adapted adjuvant therapy following surgery. While complete and permanent healing after primary surgery and appropriate adjuvant therapy seems to be possible in the majority of SGM cases, it cannot be predicted with certainty. Patients should be aware of potential progression even after a long period of freedom from disease and undergo regular checkups for any symptoms or signs of progression; as only provided most early intervention, patients are unlikely to die from LRR and metastases of their SGM tumor. As distant metastases might be detected after more than 5 years and LRRs even after 10 years, adherence to continuous long‐term follow‐up is highly recommended and prevents immature deaths as demonstrated here.

The outcome and prognosis of SGM patients benefit from availability [^18^F]‐FDG‐PET/CT imaging and introduction of regular use of [^18^F]‐FDG‐PET/CT for head and neck cancers in general as this is associated with higher sensitivity and more accurate diagnostics, especially in cases of suspected distant metastasis. We have seen more distant metastasis since 2007, presumably due to more accurate diagnostics and frequent use of [^18^F]‐FDG‐PET/CT imaging along with more frequent detection of clinicopathologic features associated with DM (small SGMs, T3‐T4, N2b upwards, ENE, and G3‐G4) since 2007.^21^ However, we cannot exclude the possibility that changing environments have contributed to increased numbers of SGM and SGM with distant metastases (M1) at time of diagnosis. Interestingly, the heightened frequency of distant metastasis did not increase cancer‐related mortality or impair OS, whereas conditional OS and especially DSS was numerically improved. Entity‐dependent risk analyses with regard to outcome data are necessary in order to be able to assign prognostic clinical and pathological risk factors to histological subgroups.

It should be acknowledged that our current treatment modalities may incur iatrogenic post‐morbidities, potentially diminishing patients' life expectancy due to systemic long‐term adverse effects (secondary malignancies, distant metastasis, leukocyte abnormalities, psychosocial sequelae, cardiopulmonary complications, postoperative complications, and reduced quality of life). This raises the question of potential over‐diagnostics and overtreatment despite limited therapeutic options. The literature indicates a risk of secondary cancers following a primary salivary gland tumor.^22^, ^23^, ^24^ Therefore, the treatment regimen, particularly cisplatin‐based ChT and radiotherapy, must be discussed as a potential risk factor for the development of distant metastasis^25^ and second malignancies. Current primary interventions may lack sufficient targeting precision.

### 4.3 Strength and limitations

By including the whole number of patients with pathologic confirmed SGM treated in our certified tumor center, our retrospective study reflects actual clinical practice. As SGM patients had regular and close follow‐up visits for up to 120 months we were able to gather continuously the relevant information that was recorded in electronic health records and, since 2013, in proprietary software (OncoFunction) as long as patients underwent tumor‐specific follow‐up in domo. We linked treatment results to quality improvement measures such as MDTB, [^18^F]‐FDG‐PET/CT, and IMRT in a comparatively large patient cohort.

Censorship due to loss to follow‐up was applied to patients who underwent tumor‐specific follow‐up at peripheral healthcare providers closer to their place of residence. The referral area is correspondingly large due to the expertise available, so that peripheral late follow‐up care for low‐risk tumors contributed to and in particular explains lost to follow‐up rates.

Besides several strengths of our analyses, we have to face inherited weaknesses of retrospective analyses such as attrition bias, selection bias for particular diagnostics and treatment modalities, and changes over time in factors not considered in our analyses. Patients from TI1 have a significantly greater potential follow‐up. Therefore, an inherent bias is present, particularly with regard to late tumor‐associated events, as patients diagnosed in TI2 may experience potential events in the future. Assuming that further distant metastases will be detected in TI2 patients in the future, we expect that hypothetically eradicating the bias would strengthen our hypothesis regarding DMFS. It should also be noted that TI1 patients benefit from more accurate DM diagnostics from 2007 onwards. Cutting off the follow‐up of patients from TI1 on December 31, 2006 also shows a significant improvement in LRRFS as the improvement shown occurs early on. Comparing censoring after cutting follow‐up to the time without standardized diagnostics revealed identical follow‐up times and conditional survival in both cohorts, TI1 and TI2. We acknowledge that causal inference about MDTB effectiveness is not possible with this retrospective design. We observed a more frequent diagnosis of distant metastasis that may have reversed our success in local and locoregional control. However, impaired DMFS is clearly linked to timely treatment and consequently reduced mortality of patients as DSS and OS were improved. In the absence of postmortem examination (autopsy) in most deceased SGM patients, we cannot exclude that they might have died with locoregional recurrences or distant metastases present, especially until the introduction of [^18^F]‐FDG‐PET/CT at the end of 2006. However, based on the death certificates, SGM was declared the cause of death in only five patients since 2007. There might have been documentation errors when declaring the actual cause of death on the death certificate without autopsy, etc. We hence may have been subject to a documentation bias regarding DSS, but the strength of this effect could have been even more relevant and of higher impact until 2006. For the survival outcome data on OS, LRRFS, and PFS, there might be an inflation of type 1 errors due to multiple testing. However, we show the underlying analyses for NCRS, DSS, LRFS, NRFS, and DMFS individually, so that no Bonferroni correction or Benjamini–Hochberg procedure is necessary for the interpretation of the results but can be easily done. This applies equally to type 2 error inflation. We think our work is transferable to other certified cancer centers for head and neck tumors and underlines the need for referral to specialized clinics by general practitioners and dentists. Patients should undergo standardized follow‐up at these centers for at least 10 years. We were able to show that reduced DMFS since 2007 can only be partially interpreted as an artifact linked to enhanced diagnostics through implementation of [^18^F]‐FDG‐PET/CT. Standardized diagnostic and therapeutic algorithms and higher sensitivity of imaging have contributed to significantly higher detection of distant metastasis (RR DM in TI2 2.149, 95% CI [1.033–4.473]; *p* = .038). However, poorer DMFS seems to be not a sign of poorer care in general as early detection of relapse and distant metastases in particular may increase the chance of timely intervention and achieving the ultimate goal of precision medicine, delivering the right treatment to the right patient at the right time, all the time. This is supported by the analyses of conditional survival after detection of M1/rM1 since 2007. As only *n* = 49 patients enter the analyses of conditional survival after detection of M1/rM1, the respective findings might be underpowered. However, a deterioration in DM‐specific tumor care does not correspond to the statistical trend that we observed and suggests that a larger population would rather support our interpretation regarding DM‐specific tumor care. Nevertheless, in view of over time increasing numbers of more aggressive SGM tumors, there should be more research for the treatment of SGM in order to be able to observe an improvement in OS and NCRS at some point.

## 5 CONCLUSIONS

Our data shows an evaluation of multidisciplinary decision making in the tumor board with orientation to international guidelines since 2007 for rare salivary gland carcinomas over an observation period of 34 years at a certified tumor center. Regular assessment of (eventually changing) organ function during dedicated consultation hours offered from specialized ENT surgeons was linked to early diagnosis of relapse and utilization of [^18^F]‐FDG‐PET/CT imaging. Prognostic factors for distant metastasis confirmed in the literature have increased in TI comparison, therefore suggesting further impact on reduced DMFS. The standardized treatment, however, coupled with appropriate decision making by the multidisciplinary team for adequate treatment and surveillance especially improved the LRRFS, while DSS of patients only improved numerically. Our procedures led to a reduced number of cancer‐related deaths, so that detection of distant metastases of salivary gland carcinomas rarely has to be considered as a death sentence. So far, it remains challenging to explain why better locoregional control does not provide an OS advantage.

## AUTHOR CONTRIBUTIONS

**Nils Feldmann:** Conceptualization; investigation; funding acquisition; writing – original draft; methodology; validation; visualization; writing – review and editing; software; formal analysis; project administration; data curation; resources. **Gunnar Wichmann:** Conceptualization; writing – original draft; investigation; funding acquisition; methodology; validation; visualization; writing – review and editing; software; formal analysis; project administration; data curation; supervision; resources. **Andreas Dietz:** Conceptualization; writing – original draft; validation; writing – review and editing; supervision. **Markus Pirlich:** Conceptualization; investigation; funding acquisition; writing – original draft; methodology; validation; visualization; writing – review and editing; formal analysis; project administration; data curation; supervision; resources.

## CONFLICT OF INTEREST STATEMENT

The authors declare no conflict of interest.

## ETHICS STATEMENT

The ethics committee of the University of Leipzig approved this study based on the Declaration of Helsinki with the votes 201‐10‐12,072,010 and 202‐10‐12,072,010. All participating patients have provided their written informed consent to participate in this study.

## Data Availability

The data that support the findings of this study are available from the corresponding author upon reasonable request.

## References

[1] WHO Classification of Tumours Editorial Board . Head and Neck Tumours. WHO Classification of Tumours Series. Vol 9. 5th ed. International Agency for Research on Cancer; 2022.

[2] Alsanie I , Rajab S , Cottom H , et al. Distribution and frequency of salivary gland tumours: an international multicenter study. Head Neck Pathol. 2022;16(4):1043‐1054. doi:10.1007/s12105-022-01459-0 35622296 PMC9729635

[3] Varoquaux A , Fakhry N , Baujat B , et al. Diagnostic imaging of salivary gland cancers: REFCOR recommendations by the formal consensus method. Eur Ann Otorhinolaryngol Head Neck Dis. 2024;141(1):27‐31. doi:10.1016/j.anorl.2023.11.007 38036312

[4] Sood S , McGurk M , Vaz F . Management of salivary gland tumours: United Kingdom national multidisciplinary guidelines. J Laryngol Otol. 2016;130(S2):S142‐S149. doi:10.1017/S0022215116000566 27841127 PMC4873929

[5] van Herpen C , Vander Poorten V , Skalova A , et al. Salivary gland cancer: ESMO‐European reference network on rare adult solid cancers (EURACAN) clinical practice guideline for diagnosis, treatment and follow‐up. ESMO Open. 2022;7(6):100602. doi:10.1016/j.esmoop.2022.100602 36567082 PMC9808465

[6] Geiger JL , Ismaila N , Beadle B , et al. Management of salivary gland malignancy: ASCO guideline. J Clin Oncol. 2021;39(17):1909‐1941. doi:10.1200/JCO.21.00449 33900808

[7] Courtade‐Saïdi M , Uro‐Coste E , Vergez S , et al. Cytopathological analysis of salivary gland cancer: REFCOR recommendations by the formal consensus method. Eur Ann Otorhinolaryngol Head Neck Dis. 2024;141(2):87‐91. doi:10.1016/j.anorl.2023.11.002 38052703

[8] Vergez S , Chabrillac E , Fakhry N . Salivary gland cancer: recommendations by formal consensus, for the French network of rare head and neck tumors (REFCOR). Eur Ann Otorhinolaryngol Head Neck Dis. 2024;141(1):3‐4. doi:10.1016/j.anorl.2023.11.012 38092570

[9] Wichmann G , Pavlychenko M , Willner M , et al. Standardized diagnostic workup and patient‐centered decision making for surgery and neck dissection followed by risk‐factor adapted adjuvant therapy improve loco‐regional control in local advanced oral squamous cell carcinoma. Front Oncol. 2021;11:737080. doi:10.3389/fonc.2021.737080 34868927 PMC8636007

[10] Barry B , Verillaud B , Jegoux F , et al. Surgery of major salivary gland cancers: REFCOR recommendations by the formal consensus method. Eur Ann Otorhinolaryngol Head Neck Dis. 2024;141(3):153‐160. doi:10.1016/j.anorl.2023.11.005 38040591

[11] Pham Dang N , Jegoux F , Barry B , et al. Surgery of sublingual and minor salivary gland cancer: REFCOR recommendations by the formal consensus method. Eur Ann Otorhinolaryngol Head Neck Dis. 2023;141:335‐338. doi:10.1016/j.anorl.2023.11.011 38052702

[12] Thariat J , Ferrand F‐R , Fakhry N , et al. Radiotherapy for salivary gland cancer: REFCOR recommendations by the formal consensus method. Eur Ann Otorhinolaryngol Head Neck Dis. 2023;141:221‐226. doi:10.1016/j.anorl.2023.11.006 38030445

[13] Baujat B , Vergez S , Jegoux F , et al. Lymph node surgery for salivary gland cancer: REFCOR recommendations by the formal consensus method. Eur Ann Otorhinolaryngol Head Neck Dis. 2023;141:215‐220. doi:10.1016/j.anorl.2023.11.001 38036313

[14] Yan F , Lao WP , Nguyen SA , Sharma AK , Day TA . Elective neck dissection in salivary gland malignancies: systematic review and meta‐analysis. Head Neck. 2022;44(2):505‐517. doi:10.1002/hed.26923 34862810

[15] Lau VH , Aouad R , Farwell DG , Donald PJ , Chen AM . Patterns of nodal involvement for clinically N0 salivary gland carcinoma: refining the role of elective neck irradiation. Head Neck. 2014;36(10):1435‐1439. doi:10.1002/hed.23467 24038533

[16] Ferrand F‐R , Even C , Chabrillac E , et al. Systemic therapies for salivary gland cancer: adenoid cystic carcinoma. REFCOR recommendations by the formal consensus method. Eur Ann Otorhinolaryngol Head Neck Dis. 2023;141:286‐291. doi:10.1016/j.anorl.2023.11.009 38061943

[17] Sarradin V , Digue L , Vergez S , et al. Systemic therapies for salivary gland carcinoma (excluding adenoid cystic carcinoma): REFCOR recommendations by the formal consensus method. Eur Ann Otorhinolaryngol Head Neck Dis. 2023;141:280‐285. doi:10.1016/j.anorl.2023.11.004 38040592

[18] Mueller SK , Haderlein M , Lettmaier S , et al. Targeted therapy, chemotherapy, immunotherapy and novel treatment options for different subtypes of salivary gland cancer. J Clin Med. 2022;11(3):720. doi:10.3390/jcm11030720 35160172 PMC8836387

[19] Nachtsheim L , Mayer M , Meyer MF , et al. Incidence and clinical outcome of primary carcinomas of the major salivary glands: 10‐year data from a population‐based state cancer registry in Germany. J Cancer Res Clin Oncol. 2023;149:3811‐3821. doi:10.1007/s00432-022-04278-6 35994118 PMC10314868

[20] Guntinas‐Lichius O , Wendt TG , Buentzel J , et al. Incidence, treatment, and outcome of parotid carcinoma, 1996–2011: a population‐based study in Thuringia, Germany. J Cancer Res Clin Oncol. 2015;141:1679‐1688. doi:10.1007/s00432-015-1961-y 25800621 PMC11824054

[21] Nam SJ , Roh JL , Cho KJ , Choi SH , Nam SY , Kim SY . Risk factors and survival associated with distant metastasis in patients with carcinoma of the salivary gland. Ann Surg Oncol. 2016;23:4376‐4383. doi:10.1245/s10434-016-5356-3 27338749

[22] Megwalu UC , Shin EJ . Second primaries after major salivary gland cancer. Otolaryngol Head Neck Surg. 2011;145:254‐258. doi:10.1177/0194599811402899 21493280

[23] Hirvonen K , Rantanen M , Haapaniemi A , Pitkäniemi J , Malila N , Mäkitie AA . Second primary cancer after major salivary gland carcinoma. Head Neck. 2018;40:251‐258. doi:10.1002/hed.24937 28960648

[24] Feng Y , Qian K , Guo K , Shi Y , Zhou J , Wang Z . Effectiveness and risk of second primary malignancies after radiotherapy in major salivary gland carcinomas: a retrospective study using SEER database. Head Neck. 2024;46(5):1201‐1209. doi:10.1002/hed.27664 38284127

[25] Zhang Y , Shi G , Zhang H , et al. Dexamethasone enhances the lung metastasis of breast cancer via a PI3K‐SGK1‐CTGF pathway. Oncogene. 2021;40:5367‐5378. doi:10.1038/s41388-021-01944-w 34272474 PMC8413128

